# Chaperone‐mediated autophagy: Molecular mechanisms, biological functions, and diseases

**DOI:** 10.1002/mco2.347

**Published:** 2023-08-30

**Authors:** Ruchen Yao, Jun Shen

**Affiliations:** ^1^ Division of Gastroenterology and Hepatology Key Laboratory of Gastroenterology and Hepatology Ministry of Health, Inflammatory Bowel Disease Research Center Shanghai China; ^2^ Renji Hospital, School of Medicine Shanghai Jiao Tong University Shanghai China; ^3^ Shanghai Institute of Digestive Disease Shanghai China

**Keywords:** carcinoma, chaperone‐mediated autophagy, neurodegenerative disorders, signaling regulation, therapeutic potential

## Abstract

Chaperone‐mediated autophagy (CMA) is a lysosomal degradation pathway that eliminates substrate proteins through heat‐shock cognate protein 70 recognition and lysosome‐associated membrane protein type 2A‐assisted translocation. It is distinct from macroautophagy and microautophagy. In recent years, the regulatory mechanisms of CMA have been gradually enriched, including the newly discovered NRF2 and p38–TFEB signaling, as positive and negative regulatory pathways of CMA, respectively. Normal CMA activity is involved in the regulation of metabolism, aging, immunity, cell cycle, and other physiological processes, while CMA dysfunction may be involved in the occurrence of neurodegenerative disorders, tumors, intestinal disorders, atherosclerosis, and so on, which provides potential targets for the treatment and prediction of related diseases. This article describes the general process of CMA and its role in physiological activities and summarizes the connection between CMA and macroautophagy. In addition, human diseases that concern the dysfunction or protective role of CMA are discussed. Our review deepens the understanding of the mechanisms and physiological functions of CMA and provides a summary of past CMA research and a vision of future directions.

## Graphical abstract

Chaperone‐mediated autophagy (CMA) is a lysosomal protein degradation pathway distinct from macroautophagy and microautophagy. CMA is regulated by signaling pathways and is involved in the maintenance of physiological functions, and its dysfunction is associated with a range of diseases.

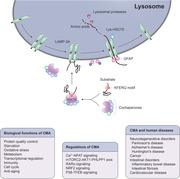

## 1 INTRODUCTION

**FIGURE 1:**
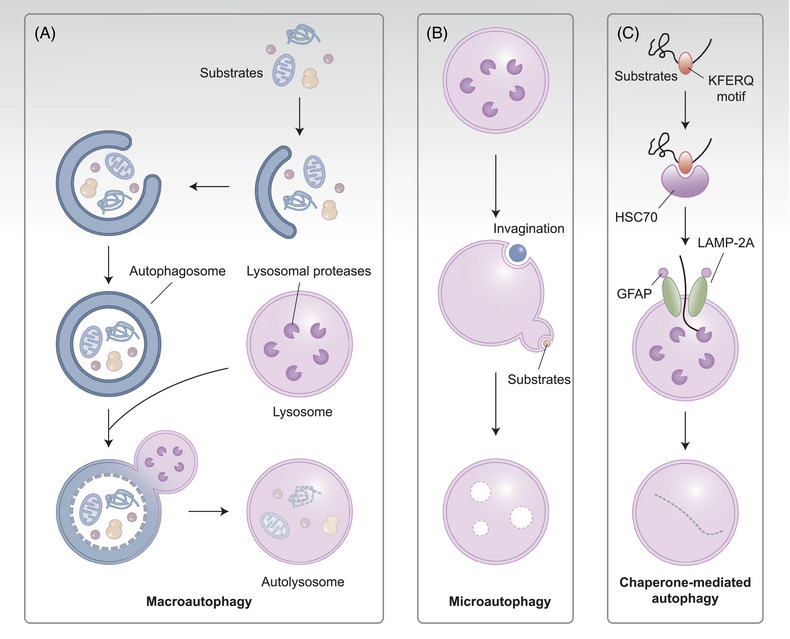
Three types of autophagy. (A) Macroautophagy: Substrates for macroautophagy (including proteins and organelles such as ribosomes and mitochondria) are first wrapped by double‐layer membranes arising from the endoplasmic reticulum, Golgi apparatus, and plasma membrane to gradually form an autophagosome. Afterward, membranes of the lysosome and autophagosome fuse to shape an autolysosome, in which the substrates are degraded via lysosomal proteases. (B) Microautophagy: First, the membrane lipid spontaneously forms a pit accompanied by a tendency to inward invaginations. Subsequently, protein substrates are progressively encapsulated by the lysosome through vesicles formed by the invagination or protrusion of the lysosomal membrane. Microautophagy derives its name from the small size of the formed vesicles compared to macroautophagy. Finally, the vesicles together with the encapsulated substrates are degraded by hydrolytic enzymes. (C) Chaperone‐mediated autophagy: The KFERQ motif of the folded protein substrates is first recognized by HSC70, and the protein then enters the lysosomal lumen in an unfolded form through LAMP‐2A on the lysosomal membrane, where it is degraded by lysosomal enzymes into amino acid.

Sixty years after the discovery of autophagy, tremendous researches have focused on the molecular mechanism and fine regulation thereof.^1^ As an important protein degradation pathway in vivo, autophagy plays its unique role, meanwhile cooperating with the ubiquitin–proteasome system, altogether finely controlling the abundance of proteins in cells to maintain proteostasis.^2^, ^3^ These two pathways were awarded Nobel Prizes in 2004 and 2016 respectively, highlighting their status in biological research. As the major pathway of protein degradation, autophagy is not a simplistic process of protein elimination, but rather a dynamic process that is carefully regulated by the degradation of specific proteins for the renewal of intracellular composition and maintenance of homeostasis.^4^ Among the three types of autophagy (i.e., macroautophagy, chaperone‐mediated autophagy [CMA], and microautophagy; Figure 1) that have been revealed so far, macroautophagy is the most widely studied while microautophagy is less understood. In contrast to macroautophagy and microautophagy, CMA does not involve the formation of vesicles. Instead, the lysosome‐associated membrane protein type 2A (LAMP‐2A) complex is assembled for the translocation into the lysosome.^5^, ^6^ Target proteins that are degraded via CMA must contain the KFERQ motif, which enables the high selectivity of CMA among the three autophagy pathways. This specificity allows CMA to play a distinct role in the breakdown of specific proteins. In addition, unlike macroautophagy which is observed in a variety of eukaryotes including yeast and zebrafish, CMA appears to be more exclusive as it has only been observed in birds and mammals.^4^ This specificity presents challenges during the study of CMA since simple models including yeast and *Caenorhabditis elegans* fail to be applied.^7^, ^8^ The uniqueness behind CMA attracts researchers to conduct further studies on its physiological processes as well as pathological processes caused by impaired CMA.

Earlier studies depicted the physiological role of CMA in stress, particularly its activation during starvation, hypoxia, and oxidative stress.^9^, ^10^, ^11^ Those findings served as a foundation for subsequent research which has yielded breakthroughs and provided new insights into the physiological functions of CMA, as well as its contributions to various diseases. For instance, this year, the inflammasome sensor protein NLR (nucleotide‐binding and oligomerization domain‐like receptor) family, pyrin domain containing 3 (NLRP3) was identified as a novel CMA substrate during palmitoylation that mediates dysregulation of innate immune responses, providing new insights for the involvement of CMA in the regulation of immune response.^12^, ^13^ Additionally, it was discovered that CMA plays a protective role in atherosclerosis in cardiovascular disease. Impaired CMA was shown to accelerate the progression of atherosclerosis by targeting macrophages and vascular smooth muscle cells (VSMCs) that are responsible for plaque formation.^14^, ^15^ Those findings enrich the understanding of protein quality regulation, as well as provide potential therapeutic targets for diseases.

In this review, we discuss the unique roles of CMA that distinguish it from macroautophagy and microautophagy. Further, we highlight the interaction between CMA and macroautophagy, as CMA does not exist independently and sometimes compensates with macroautophagy to jointly maintain protein homeostasis. We believe that this interaction deserves further investigation to help clarify the precise fit between different autophagy and develop better tools to regulate specific autophagy, which is often overlooked in reviews. Finally, we discuss the involvement of CMA in human diseases, including tumors, neurodegenerative disorders (NDs), cardiovascular diseases, and intestinal disorders which have been extensively studied.

## 2 THE UNIQUE MECHANISM OF CMA

Autophagy can be divided into three major forms: macroautophagy, microautophagy, and CMA.^16^, ^17^ In macroautophagy, an isolation membrane confines intracellular cargo such as protein aggregates, organelles, and ribosomes to form an autophagosome. Subsequently, the autophagosome fuses with the lysosome to form an autolysosome, in which the cargo is degraded by lysosomal acid proteases.^5^ Microautophagy requires random invagination of the lysosomal membrane to pack the substrates, including soluble intracellular substrates in nonselective microautophagy, and peroxisomes, nuclei, or mitochondria in selective microautophagy.^6^, ^18^ Unlike microautophagy and macroautophagy, which comprise vesicular cargo delivery modes, CMA does not rely on autophagosomes to capture substrates, nor does it engulf substrates via the invagination of the lysosomal membrane. In CMA, the translocation of the substrates into lysosomes is mediated by LAMP‐2A on the lysosomal membrane.^19^

**FIGURE 2:**
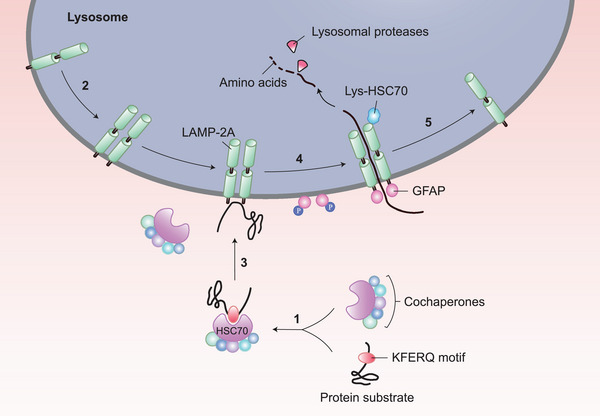
Degradation of protein substrates via CMA. (1) Identification: HSC70 recognizes the KFERQ motif of the substrate protein and forms an HSC70–substrate complex. Cochaperones, including HIP, HSP90, HSP40, HOP, and BAG‐1, are also involved in this process. (2) Assembly of LAMP‐2A: LAMP‐2A consists of a transmembrane region that is highly glycosylated and a tail in the cytoplast. LAMP‐2A is a monomeric protein. During the combination process, LAMP‐2A forms a homotrimeric complex with HSC70 and the substrate. (3) Combination: The HSC70–substrate complex binds to the C‐terminal tail of LAMP‐2A on the surface of the lysosome. Dephospho‐glial fibrillary acidic protein (GFAP) assists to maintain the stability of LAMP‐2A. (4) Translocation and degradation: The substrate translocates into the lysosome and is degraded into amino acids by proteases immediately. Those amino acids can then be transported out of the lysosome and involved in cellular metabolism, including the synthesis of new proteins. Lysosomal HSC70 (Lys‐HSC70) promotes the translocation of the substrate. (5) Disassembly of LAMP‐2A: GFAP is phosphorylated and dissociates from LAMP‐2A. Subsequently, LAMP‐2A is disassembled into the monomeric form and prepared for the next CMA process.

Both macroautophagy and microautophagy can be selective or nonselective, whereas CMA is selective for substrate recognition. The process and molecular mechanism of CMA are briefly outlined in Figure 2. Proteins that serve as substrates for CMA to be degraded by lysosomes must contain KFERQ sequences (KFERQ‐like motif‐bearing proteins). First, heat‐shock cognate protein 70 (HSC70) identifies the KFERQ sequence and delivers the substrate protein to the surface of the lysosomal membrane to form a chaperone‐substrate complex. Next, this complex binds to the C‐terminal tail of LAMP‐2A on the membrane to form a homotrimeric LAMP‐2A–substrate complex. Finally, the substrate enters the lysosome in an unfolded state and is degraded, leaving LAMP‐2A across the lysosomal membrane and HSC70 in the cytoplasm. The hydrolase decomposes the protein substrate into amino acids for protein synthesis, and LAMP2 is detached from the LAMP‐2A–HSC70 complex and relocates to the lysosomal membrane to participate in the cyclic process of CMA.^20^, ^21^, ^22^ Detached HSC70 can recognize another substrate for the next CMA.^23^ In addition to assisting in the binding of substrates and LAMP‐2A, HSC70 participates in the assembly of the LAMP‐2A–substrate complex, which maintains the stability of LAMP‐2A.^24^, ^25^ Apart from HSC70, other chaperones, including heat shock protein (HSP) 90, HSP40, HSP70–HSP90 organizing protein (HOP), HSP70‐interacting protein (HIP), and Bcl‐2‐associated athanogene‐1 (BAG‐1), which are called cochaperones, are also involved in this process to help HSC70 in different aspects.^26^ More specifically, HSP90 maintains the unfolded status of its substrates,^27^ HSP40 stimulates the ATPase activity of HSC70, HIP maintains the stability of the adenosine diphosphate (ADP) state of HSC70 to prevent premature dissociation between HSC70 and the substrate,^28^, ^29^ HOP promotes the combination of HSC70 and HSC90, and BAG‐1 may have a dual effect that positively or negatively regulates HSC70.^30^, ^31^ LAMP‐2A acts as a gatekeeper that controls the translocation of substrate proteins to the lysosomal membrane. Failure of multimerization and blocking of the C‐terminus of LAMP‐2A may lead to blockage of the CMA process since LAMP‐2A is unable to bind with HSC70 and substrates.^22^

In the last three decades, since CMA was first described, the intricate processes and molecular mechanisms underlying this unique subtype of autophagy have started being uncovered. CMA interferes with glucose and lipid metabolism by degrading key enzymes and lipid droplet‐associated proteins during glycolipid metabolism. Here, it degrades glyceraldehyde 3‐phosphate dehydrogenase (GAPDH) implicated in glucose metabolism and glycerol‐3‐phosphate dehydrogenase 2 and acyl‐coenzyme A dehydrogenase long chain implicated in lipid metabolism.^32^, ^33^, ^34^ Moreover, CMA maintains T cell activation by degrading the negative regulator Itch and the regulator of calcineurin‐1 (Rcan‐1).^35^ CMA also resists hypoxia‐mediated cell cycle arrest by degrading hypoxia‐inducible factor‐1 alpha (HIF‐1α).^36^ In some age‐related diseases, the degradation of mutated proteins by CMA is essential in antiaging.^19^ In addition, CMA helps with the rhythmic removal of clock machinery proteins to maintain the function of the clock transcriptional program.^37^ Normal functioning of CMA is involved in maintaining homeostasis in the body, while defects in CMA may be related to human diseases, including Alzheimer's disease (AD) and cancer.

## 3 BIOLOGICAL FUNCTIONS OF CMA

**FIGURE 3:**
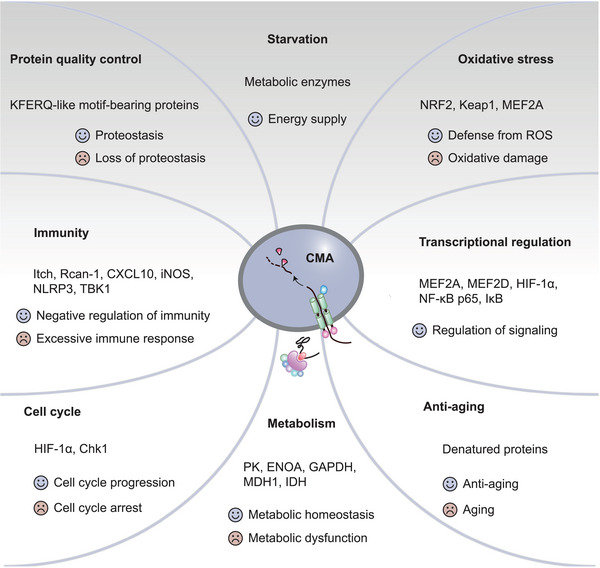
Physiological functions of CMA. CMA modulates physiological functions through the degradation of related protein substrates under different conditions, some of which are normal/denatured proteins, key enzymes in biochemical reactions, molecules in signal transduction pathways, or transcription factors that regulate gene expression. The title of each panel is the physiological role performed by CMA, followed by the CMA substrate of the process. The smiley face and the crying face represent the consequences resulting from normal and abnormal CMA function, respectively. Chk1, checkpoint kinase 1; CXCL10, chemokine (C‐X‐C) motif ligand; ENOA, α‐enolase; IDH, isocitrate dehydrogenases; iNOS, inducible nitric oxide synthase; GAPDH, glyceraldehyde‐3‐phosphate dehydrogenase; HIF‐1α, hypoxia‐inducible factor‐1 alpha; IκB, inhibitor of NF‐κB; Keap1, Kelch‐like ECH‐associated protein 1; MDH1, malate dehydrogenase‐1; MEF2, myocyte enhancer factor 2; NF‐κB, nuclear factor‐kappaB; NLRP3, NOD‐, LRR‐ and pyrin domain‐containing 3; NRF2/NFE2L2, nuclear factor, erythroid 2‐like 2; PK, pyruvate kinase; Rcan‐1, regulator of calcineurin‐1; TBK1, TANK binding kinase‐1.

CMA is involved in the regulation of various physiological activities through the degradation of protein substrates. The integrity of the CMA function guarantees the normal performance of those physiological processes, and CMA deficiency may upset this balance (Figure 3).

### 3.1 Protein quality control

Serving as the sole substrate of CMA, the protein quality is delicately regulated by CMA to maintain a balance, which is the basis for CMA to play its various physiological roles. Confronting diverse stressors including protein damage, starvation, hypoxia, and oxidative stress, CMA is activated to dispose of damaged proteins.^10^, ^11^, ^38^ This timely elimination is particularly crucial in neuronal cells with high sensitivity to misfolded proteins, as they are highly differentiated permanent cells that are unable to achieve self‐renew through mitosis. Thus, CMA is demonstrated as a critical factor in maintaining the stability of neuronal protein quality.^39^ CMA degrades proteins that are responsible for neuronal degeneration to resist the toxicity of denatured proteins.^40^, ^41^, ^42^ CMA was also found to be upregulated in response to genotoxicity, degrading checkpoint kinase 1 (Chk1) to assure nuclear proteostasis. CMA also stabilizes the MRN (Mre11–Rad50–Nbs1) complex in the DNA repair pathway and maintains genome stability.^43^

As a result of aging or disease progression, damage to the CMA may occur and consequently lead to an imbalance in protein mass.^44^ In the liver, protein mass remains stable due to compensatory macroautophagy and the action of the proteasome.^26^ However, this compensatory role has not been elucidated in the nervous system. As the NDs progress, CMA degeneration allows the degenerative protein to accumulate, leading to a series of pathological changes.^41^

Substrates for CMA include not only abnormally synthesized and damaged proteins but also normal proteins. Normal proteins which contain KFERQ‐motif are folded to act as CMA substrates, thereby regulating the signaling pathways in which these proteins are involved.^22^, ^45^ For example, during adipogenesis, CMA degrades limiting proteins in multiple crucial steps to drive adipogenesis.^46^

### 3.2 Starvation

Starvation was of key importance in the discovery and establishment of CMA. In 1978, Dice^47^ was the first to discover that degradation rates of cytoplasmic proteins in diabetic and starved rats vary. Subsequently, KFERQ sequences were identified from these faster degrading proteins, leading to the determination of the selectivity of CMA.^48^ Before the widespread use of gene knockout technology, deprivation of serum or food to induce starvation was a considered method to enhance CMA activity in cell or animal models in research.

In general, short‐term starvation first activates macroautophagy, whereas CMA is activated when starvation lasts longer than 10 h.^49^ In fibroblast models, stimulation of β‐hydroxybutyrate can stimulate CMA by oxidizing substrates to degrade GAPDH and ribonuclease A.^50^, ^51^ In the starved state, the rate of glycolysis decreases, leading to increased CMA degradation of GAPDH and aldolase‐B which serve as key enzymes of glycolysis to replenish proteins required for gluconeogenesis.^49^ CMA was also found to be upregulated in the model of neurotoxicity induced by paraquat when implementing prolonged intermittent fasting.^52^ In mice models, lifted CMA promoted by fasting resulted in decreased apoptosis and reduced accumulation of amyloid precursor protein (APP) in neurodegenerative regions.^52^ Therefore, it is speculated that caloric reduction in simulated starvation may be a potential therapeutic tool for NDs represented by AD.

### 3.3 Oxidative stress

The role of macroautophagy in reactive oxygen species (ROS) stress has been elucidated. ROS induce macroautophagy, which reduces tissue damage by degrading oxidative products. Multiple ROS signaling pathways induce macroautophagy, including ROS‐FOXO3‐LC3/BNIP3, ROS‐NRF2 ‐P62, ROS‐HIF1‐BNIP3/NIX, and ROS‐TIGAR.^53^ In addition to macroautophagy, CMA has been reported to be involved in repair processes following ROS damage.

During oxidative stress, CMA is activated to degrade oxidized proteins in mouse fibroblasts; however, the details of the process and specific proteins involved, have not been elucidated. Under stress conditions induced by CMA, increased LAMP‐2A levels are caused by reduced degradation and relocation from the lumen to the membrane, rather than by the synthesis of new proteins.^54^ In ROS, de novo synthesis accounts for increased LAMP‐2A.^55^ LAMP‐2A and CMA activity increase during oxidative stress, resulting in the clearance of oxidized proteins. Nevertheless, the specific mechanism of CMA activation is not well understood. ROS activate CMA through nuclear factor, erythroid 2‐like 2 (NRF2, also known as NFE2L2), an essential signal transduction molecule in oxidative stress, binding to the antioxidant response element (ARE) in the promoter region to regulate the antioxidant defense function of cells.^56^ In turn, CMA can also regulate NRF2 levels, and the substrate in this process is Kelch‐like ECH‐associated protein 1 (Keap1), which promotes the degradation of NRF2.^57^, ^58^ Thus, the functional integrity of CMA can maintain NRF2 activity by decreasing Keap1 levels to ensure the cellular response to antioxidant damage.^57^ Indeed, CMA inhibition is sufficient to enable increased cell death in rat liver cells under menadione‐induced oxidative damage, although macroautophagy is considered to be the main modality of resistance to this oxidative stress.^59^ Different from the overactivation of c‐Jun N‐terminal kinase/c‐Jun signaling in cell death induced by macroautophagy, cell death arising from CMA inhibition attributes to caspase‐dependent apoptosis. However, the detailed mechanism remains to be further investigated.^59^

Since oxidative stress constitutes an important link in various diseases, the regulation of CMA is involved in the process of oxidative damage caused by certain diseases. In a mouse model in which the spinal cord was hemitransected, histone deacetylase‐6 (HDAC6) regulates CMA to prevent oxidative damage in injured neurons after experimental spinal cord injury, and oxidative stress damage is exacerbated when HDAC6 or CMA is inhibited. the upregulation of HDAC6 via CMA is mediated by HSP90 deacetylation, which is one of the cochaperones described previously.^60^ However, the substrate for this process is not well known. *PARK7*, a gene for familial PD, expresses a PARK7 protein that plays a pivotal role in antioxidation, and abnormal function will lead to mitochondrial damage, and CMA was found to be a major regulatory pathway of PARK7 in 1‐methyl‐4‐phenylpyridinium (MPP)‐induced oxidative damage.^61^, ^62^, ^63^ In a mouse neuronal cell model, CMA degrades the incompetent PARK7 protein to maintain the integrity of mitochondrial morphology and function. In contrast, it was found a decreased potential of mitochondrial membrane and broken mitochondria in the LAMP‐2A knockout cell model.^63^

Nevertheless, the regulatory capacity of CMA in oxidative stress is limited, and excessive oxidative stress can dampen CMA function. As an example, Myocyte enhancer factor 2 A (MEF2A) protects primary neurons from oxidative stress damage, and CMA is an important degradation pathway for MEF2A.^64^ In the initial stage of oxidative stress, CMA activity is elevated to sustain MEF2A homeostasis. However, excessive oxidative stress makes the reduced CMA activity unable to degrade MEF2A, leading to the accumulation of incompetent MEF2A, reduced binding capacity to DNA, and ultimately oxidative stress damage to neurons.^65^

### 3.4 Metabolism

CMA regulates metabolism by degrading enzymes involved in glycolipid metabolism, and liver CMA‐deficient mice developed abnormalities in glycolipid metabolism and even systemic metabolism. In liver‐specific LAMP‐2A knock‐out mice, blockage of CMA alone was observed to be sufficient to depress the capacity of glycogen storage and gluconeogenesis and enhance glycolysis in hepatocytes.^33^ This effect is caused by the accumulation of CMA substrates during gluconeogenesis, including pyruvate kinase, α‐enolase, GADPH, and malate dehydrogenase‐1, which promote glycolysis.^33^

About 30% of CMA substrates are involved in lipid metabolism, including triglyceride synthesis and lipid binding and transportation. CMA deficiency mediated by LAMP‐2A knockdown leads to a rise in lipogenesis‐promoting enzymes including GADPH and accumulation of cholesterol ester and triacylglyceride, and ultimately mediates steatosis in the mouse liver. Worse still, the primary abnormality of lipid metabolism in the liver is sufficient to cause systemic metabolic and endocrine alterations in mice, including increased energy expenditure and increased adiposity with high‐fat food feeding.^33^ In the process of adipogenesis, CMA is necessary for preadipocyte differentiation. Elevated levels of LAMP‐2A were detected both at the cellular level and in mice, suggesting increased CMA activity. Among them, MYC and transforming growth factor‐β (TGFβ) were identified as the major CMA substrates during adipose differentiation.^46^

CMA was found to be engaged in the self‐renewal and differentiation of embryonic stem cells by regulating their metabolism. CMA degrades isocitrate dehydrogenases to reduce the generation of α‐ketoglutarate,^66^ which maintains the pluripotency of embryonic stem cells.^67^ Prior to embryonic stem cell differentiation, CMA activity is inhibited by pluripotency factors OCT4 (octamer‐binding transcription factor 4) and SOX2 (sex‐determining region Y‐box2), which may act as a regulatory trigger for embryonic stem cells to maintain their pluripotency.^66^, ^68^ Hence, stimulation or inhibition of CMA in embryonic stem cells may interfere with their proliferation toward differentiation or maintenance of pluripotency.

### 3.5 Transcriptional regulation

Several transcriptional regulators are substrates of CMA, such as MEF2D, which is required for neuronal survival^69^; MEF2A, which protects primary neurons from oxidative stress^65^; and HIF‐1α, which is upregulated in response to hypoxia.^70^, ^71^ CMA regulates the relevant signaling pathways by modulating the levels of these transcription factors. Since these signals are involved in the regulation of oxidative stress and the cell cycle, they will be detailed in the corresponding panels.

It is notable that CMA has been found to be involved in the regulation of a complex pathway, NF‐κB. Five transcription factors of NF‐κB bind to target DNA sequences and perform immune regulation in multiple ways, including the development of the immune system, innate immunity, adaptive response, and inflammation.^72^, ^73^ In malignant tumors, the NF‐κB pathway exerts its antitumor role, while its overactivation might promote cancer development and progression.^74^ CMA regulates the NF‐κB pathway by degrading two signaling molecules that are known to be related. The first is p65, which is the key factor in NF‐κB signaling, with impaired p65 degradation via CMA leading to exaggerated NF‐κB signaling. HSC70 recognizes and binds to the amino‐terminal reL homology domain (RHD) of p65 to form an HSC70–p65 complex which is transported to the lysosome and degraded after binding to LAMP‐2A. The degradation of p65 is mediated by CMA instead of macroautophagy because the level of p65 remains stable after the use of 3‐methyladenine (3‐MA), a macroautophagy inhibitor. Impaired CMA leads to reduced p65 degradation, thereby exaggerating NF‐κB signaling, and is related to epithelial‐to‐mesenchymal transition (EMT) progression in epithelial cells.^75^ Another CMA substrate identified for this process is IκB (inhibitor of NF‐κB). In Chinese hamster ovary cells with serum nutrient depletion, the production of ROS may mediate the degradation of the long‐lived pool of IκB by CMA. This process can be completely blocked by CMA inhibitors, suggesting that the lysosomal pathway is the predominant route of IκB degradation in this condition. Increased CMA activity is also accompanied by elevated sensitivity of NF‐κB to typical stimuli such as lipopolysaccharide (LPS), interleukin‐1 (IL‐1), and tumor necrosis factor‐alpha (TNF‐α).^76^ Still, under most conditions, IκB degradation is mainly achieved by the proteasome system after phosphorylation or ubiquitination, thus the holistic regulation of this signal by CMA needs further study.^77^, ^78^ In different stimulatory conditions (oxidative stress or starvation), CMA exerts different (inhibitory or facilitative) effects on the NF‐κB pathway, which reflects the complex regulation of this process. Accordingly, CMA functions as a regulator of the signal transduction pathway via degrading NF‐κB components or inhibitors under different stresses.

Interestingly, while CMA regulates signal transcription, it is also under the regulation of transcription factors. For instance, transcription factor EB (TFEB) promotes CMA in ovarian cancer and PD; NRF2 promotes CMA in oxidative stress; and CMA is negatively regulated by OCT4 and SOX2 in embryonic stem cells.^66^, ^79^, ^80^, ^81^

Among all of these described regulatory modalities, the mechanism is the decrease in the expression of LAMP‐2A, a key rate‐limiting step of CMA. Associated transcription factors bind at different sites of the *LAMP‐2A* promoter, thus modulating the transcription of *LAMP‐2A*.

### 3.6 Immunity

**FIGURE 4:**
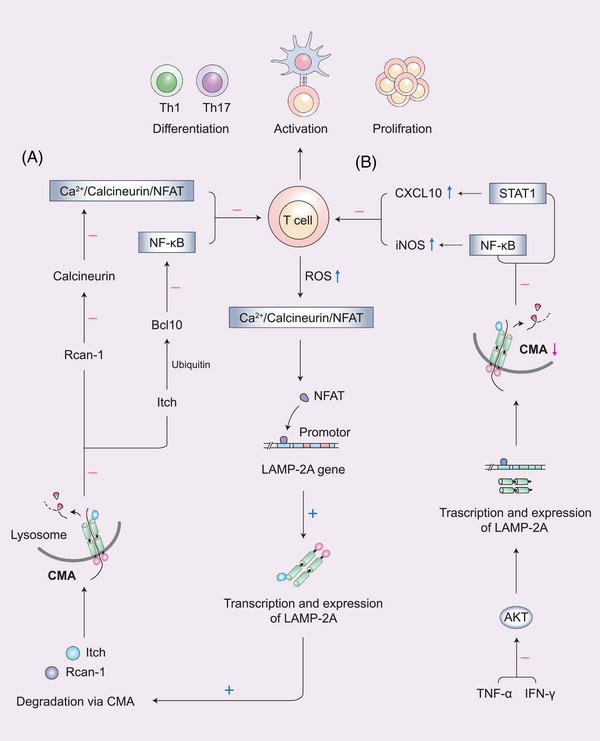
CMA sustains CD4^+^ T cell activation. (A) Degradation of Itch and Rcan‐1 via CMA maintains CD4^+^ T cell activation. Itch and Rcan‐1, both containing KFERQ motifs, are negative regulators of TCR downstream signaling pathways by inhibiting NF‐κB and Ca^2+^/Calcineurin/NFAT signaling. Itch inhibits NF‐κB signaling via ubiquitination of Bcl10, and Rcan‐1 inhibits Ca^2+^/calcineurin/NFAT signaling by reducing Calcineurin. After the activation of CD4^+^ T cells, ROS levels increase, and the Ca^2+^/calcineurin/NFAT signaling pathway becomes activated. Nuclear factor of activated T‐lymphocytes binds to the proximal promoter of *LAMP‐2A* (the specific site is unclear) to promote the transcription and expression of LAMP‐2A. Increased LAMP‐2A expression leads to increased CMA activity, which facilitates the degradation of Itch and Rcan‐1 via CMA, thereby downregulating Itch and Rcan‐1 levels to sustain CD4^+^ T cell activation and promote its differentiation of Th1 and Th17 cells. (B) Inhibition of CMA induces MSCs to suppress T cell proliferation. In the inflammatory microenvironment induced by TNF‐α and IFN‐γ, AKT signaling is activated to downregulate the transcription and expression of LAMP‐2A. Reduced CMA activity increases CXCL10 via STAT1 signaling to recruit CD4^+^ T cells, and boosts iNOS levels via NF‐κB signaling to inhibit the proliferation of T cells.

Normal T cell activation ensures the implementation of its immune effects, whereas overactivation may induce autoimmune diseases.^82^ In the comprehensive network of T cell regulation, CMA maintains CD4^+^ T cell activation by degrading Itch and Rcan‐1 (Figure 4). Moreover, the inhibition of CMA promotes the immunosuppression of mesenchymal stem cells (MSCs).^35^, ^83^ After CD4^+^ T cell activation, the increased production of ROS by mitochondria activates the Ca^2+^/calcineurin/nuclear factor of activated T‐lymphocyte (NFAT) signaling.^84^ The latter binds to the proximal promoter of *LAMP‐2A* to increase its transcription. The increased activity of cytoplasmic CMA was detected by KFERQ‐PA‐Mcherry‐1, together with reduced levels of Itch and Rcan‐1.^35^ Itch and Rcan‐1 are negative regulators of the T‐cell receptor (TCR) signaling pathways. Itch, an E3 ubiquitin ligase, terminates the activation of NF‐κB signaling downstream of TCRs by ubiquitinating Bcl10, thereby blocking the role of NF‐κB in promoting T cell proliferation and maintaining T cell survival.^85^, ^86^ A lack of itch leads to autoimmune diseases owing to overactive T cells.^87^ Rcan‐1 is an inhibitor of calcineurin, which suppresses the activation and proliferation of T cells and the differentiation of T‐helper 1 (Th1) and Th17 cells by decreasing Ca^2+^/Calcineurin/NFAT signaling pathway activity.^88^, ^89^ Both Itch and Rcan‐1 contain the KFERQ motif and serve as substrates for CMA. Increased CMA promotes the degradation of Itch and Rcan‐1 and maintains the activation of CD4^+^ T cells. In the LAMP‐2A‐deficient CD4^+^ T cell model, Itch and Rcan‐1 levels were found to increase, along with reduced T cell proliferation and proinflammatory cytokine production.^35^

In addition to protection against T cell activation, it was found that inhibition of CMA can induce MSCs to suppress T cell proliferation in the inflammatory microenvironment. TNF‐α and interferon‐gamma (IFN‐γ) inhibit CMA in MSCs through v‐akt murine thymoma viral oncogene homolog (AKT) activation, leading to decreased transcription and expression of LAMP‐2A.^83^ Impaired CMA increases chemokine (C‐X‐C) motif ligand (CXCL10) and inducible nitric oxide synthase (iNOS) levels via the activation of STAT1 and NF‐κB signaling, thus recruiting CD4^+^ T cells and inhibiting the proliferation of T cells.^83^, ^90^ MSCs are available for inflammatory diseases owing to their multi‐directional differentiation ability and powerful immune suppression.^91^ CMA‐deficient MSCs were found to reduce inflammation and improve the efficacy of an inflammatory liver injury model compared with selective cell retention‐MSCs.^92^, ^93^

Another discovery in the regulation of immune response by CMA is the degradation of NLRP3, a cytosolic signaling receptor that regulates innate immunity via the formation of inflammasomes which finally induces pyroptotic cell death.^94^ NLRP3 was identified to possess four KFERQ‐like motifs, and ZDHHC12 functions as an S‐acyltransferase, mediates palmitoylation, and promotes recognition of NLRP3 by HSC70 and subsequent NLRP3 degradation within the lysosome via CMA. This process may regulate NLRP3 which is overactivated in inflammation and CMA defects may be associated with abnormal palmitoylation that leads to auto‐inflammatory responses.^12^, ^13^

TANK binding kinase‐1 (TBK1) is a typical serine‐threonine kinase, known for its regulation of IFN‐1 production in innate immunity after infection.^95^ Previously, the major lysosomal degradation pathways of TBK1 were considered to be mitophagy and antibacterial autophagy (xenophagy), until the recognition of its KFERQ‐like motif (KFDKQ), which indicates a new pathway that regulates TBK1 signaling.^96^ Deubiquitinating enzyme ubiquitin specific peptidase 19 (USP19) facilitates CMA degradation of TBK1 rather than proteasome‐dependent degradation, thus playing a negative regulatory role in the cellular antiviral response. After CMA inhibition, IFN‐1 production was increased and the antiviral response was enhanced in mouse macrophages.^97^ Up to this point, TBK1 has been found to be regulated by phosphorylation, ubiquitination, and lysosomal pathways, all of which are regulated jointly to ensure an adequate immune response while preventing an excessive innate antiviral response. However, the interactions of these pathways have been far too poorly studied.^98^, ^99^

### 3.7 Cell cycle

HIF‐1α mediates the adaptive response to cellular hypoxia, and its overexpression arrests cells in the G1 phase.^100^ CMA, rather than macroautophagy, was identified as the primary lysosomal degradation pathway for HIF‐1α, as pharmacological inhibition or promotion of CMA elevated or decreased HIF‐1α levels, while its levels did not change measurably after inhibition of macroautophagy.^100^ HSC70 binds to the amino acid residue site of HIF‐1α and subsequently colocalizes with LAMP‐2A in the lysosome. Thus, CMA is an essential negative regulator of HIF‐1α under hypoxic stress, with the main regulator as TFEB, and CMA inhibition induces cell cycle arrest.^36^, ^70^ This pathway was confirmed in several cell lines and thus is probably a general regulatory modality.^70^

Another substrate of CMA in its regulation of the cell cycle is Chk1, an essential cell cycle checkpoint effector molecule that mediates cell cycle arrest in the S‐G2 phase by reducing activation of the downstream molecule cell cycle protein‐dependent kinase 2.^101^, ^102^, ^103^ When DNA double‐strand breaks were induced by etoposide in mouse fibroblasts, CMA was found to upregulate and degrade Chk1 in time to disengage repair proteins and restore cell cycle progression. Chk1 nuclei accumulate in CMA‐blocked cells, leading to the reduced level of MRN (Mre11–Rad50–Nbs1) complex which is responsible for DNA damage signaling. Ultimately, the accumulation of Chk1 impedes cell cycle progression and reduces the ability of cells to survive DNA damage.^43^, ^104^

In general, CMA sustains the cell cycle by degrading cell cycle‐associated proteins in hypoxia and DNA damage. In tumors, the effects of CMA are much more complex, as both positive and negative regulators may undergo degradation as CMA substrates, and it remains to be fully explored to investigate the role of CMA overactivation or inhibition in tumor promotion.^105^

### 3.8 Antiaging

At the beginning of this century, CMA activity was discovered to decrease with age, which parallels the decrease in protein degradation rate.^106^ During aging, LAMP‐2A stability decreases and the molecular chaperone HSC73 is elevated to compensate for the reduced CMA function. Yet, this compensation is distinctly limited since LAMP‐2A is the most significant rate‐limiting step of CMA. LAMP‐2A knockout cells or animal models exhibit remarkable CMA defects, and CMA activity is persistently downregulated during aging.^22^, ^106^, ^107^ In the liver and T cells model, CMA defects can be partially replenished by macroautophagy or proteasomes, while failing to completely reverse senescence‐associated disorders of glycolipid metabolism.^26^, ^108^, ^109^ Under the inflammatory environment, CMA and macroautophagy in T cells are mutually compensated, that is, a defect in either is accompanied by an upregulation of the other.^109^ However, this compensation in retinal neurodegeneration is not bidirectional. CMA compensates for macroautophagy that declines with age, while inhibition of CMA in this context does not activate macroautophagy.^110^ Thus, the role of CMA in the antiaging process in certain tissues may be irreplaceable.

The effect of CMA in degrading denatured proteins to preserve proteostasis is particularly vital for the nervous system. In the early stages of Huntington's disease (HD) characterized by neurological degeneration, LAMP‐2A expression rises and CMA activity increases to degrade mutant huntingtin (mHtt) protein. However, CMA compensation in the brain declines in the advanced stage of HD, leading to irreversible progression of neurodegeneration.^111^ Nonetheless, a report performed on healthy older adults showed that during the normal aging process, levels of LAMP‐2A in the cerebrospinal fluid did not correlate significantly with either HSC70 or oxidative stress markers. Therefore, the regulation of cerebrospinal fluid or CMA in the brain during aging still needs further investigation.^112^

Another striking discovery is the involvement of CMA in the maintenance of bone marrow hematopoietic function during aging. When CMA is inhibited, glycolysis of hematopoietic stem cell (HSC) is reduced and the repopulation capability of bone marrow is decreased. Aging is accompanied by a decrease in CMA flux and an increase in depletion of HSC, including a declination in the number of HSC and the ability of reconstruction, and a rise in ROS levels. In contrast, the ability of HSC differentiation was enhanced in aged mice and humans after the use of the CMA activator, which suggests that CMA may serve as a potential therapeutic target in the antiaging process to maintain the normal function of HSC.^113^

## 4 THE CROSSTALK BETWEEN CMA AND MACROAUTOPHAGY

**FIGURE 5:**
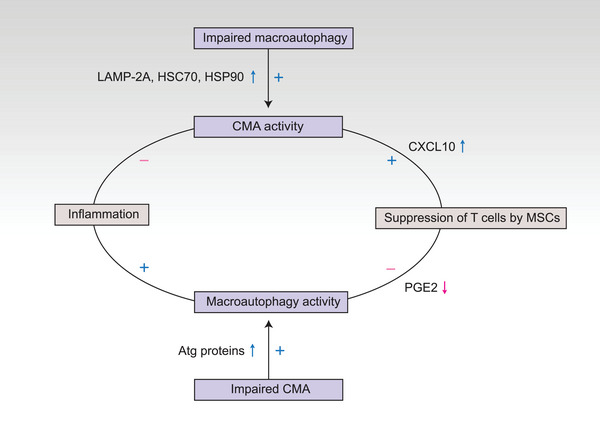
Crosstalk between CMA and macroautophagy. In inflammatory environments, CMA gets inhibited to promote MSCs‐induced T cell suppression by upregulating the level of CXCL10. Inflammation can activate macroautophagy and inhibit MSCs‐induced T cell suppression by facilitating the degradation of prostaglandin E2 (PGE2) via macroautophagy. That is, impaired CMA leads to a boost in macroautophagy activity, which suggests the crosstalk between the two subtypes of autophagy. The mechanism of increased macroautophagy in impaired CMA remains unclear; however, it has been proposed that Atg 6, 7, 8, and 16 contain the KFERQ motif, which may serve as substrates for CMA. Impaired CMA increases Atg proteins, which promotes macroautophagy. CMA also compensates for impaired macroautophagy activity. In inflammation, when macroautophagy gets inhibited, it was found the levels of LAMP‐2A, HSC70, and HSP90 are increased to induce CMA activity and maintain T cell activation.

As described above, inhibition of CMA induces MSCs to suppress T‐cell proliferation during inflammation. In addition, macroautophagy is activated during inflammation to inhibit the immunosuppression of MSCs.^109^ That is, inhibition of CMA and activation of macroautophagy led to reduced and increased T‐cell proliferation, respectively, suggesting an interaction between CMA and macroautophagy (Figure 5). Increased CMA activity has been shown in impaired macroautophagy during inflammation. Here, LAMP‐2A, HSC70, and HSP90 levels were increased, accompanied by macroautophagy inhibition. LAMP‐2A is the trigger point for CMA, and increased LAMP‐2A leads to elevated CMA activity.^54^ The mechanisms of increased LAMP‐2A depend on the stress levels. Under mild stress conditions, the de novo synthesis of LAMP‐2A is required. However, during severe stress, such as chronic starvation, the increase in LAMP‐2A is due to a slower rate of LAMP‐2A degradation. HSC70 acts as a chaperone for substrate recognition and assists in substrate degradation in CMA. The amount of HSC70 does not rise in a certain lysosome, whereas it is the increased number of lysosomes containing HSC70 that promotes CMA activity. Therefore, it is speculated that when the macroautophagy pathway is blocked, CMA activity increases and activates T cells by inhibiting the regulation of MSCs, which indicates that the compensatory CMA pathway rescues the macroautophagy pathway.

Similarly, macroautophagy can also compensate for CMA deficiency. Under stress conditions induced by starvation, macroautophagy is activated within the first 4−6 h, followed by CMA activation.^51^, ^114^ Sequential activation of macroautophagy and CMA results in the accumulation of amino acids for the synthesis of essential proteins via the degradation of nonessential proteins.^115^ Wild‐type α‐synuclein serves as a substrate for CMA, whereas mutant α‐synucleins block CMA activity and are degraded by macroautophagy.^108^ Compensatory activation of macroautophagy has also been observed in the degradation of inherent proteins in CMA‐impaired cells.^116^ As we discussed, in the inflammatory environment, impaired CMA leads to reduced T cell activation, which might induce macroautophagy to replenish immunosuppression by MSCs. Increased macroautophagy inhibits the immunosuppression of MSCs and subsequently improves T cell proliferation by increasing IL‐17 and IL‐22 levels. Although the mechanism by which macroautophagy is upregulated in CMA‐deficient cells remains unclear, the possible roles may be as follows: Atg 6, 7, 8, and 16 are detached to contain the KFERQ motif and may be substrates for CMA degradation. The reduced levels of Atg proteins caused by impaired CMA may lead to the activation of macroautophagy.^116^

## 5 SIGNALING PATHWAYS REGULATING CMA

**FIGURE 6:**
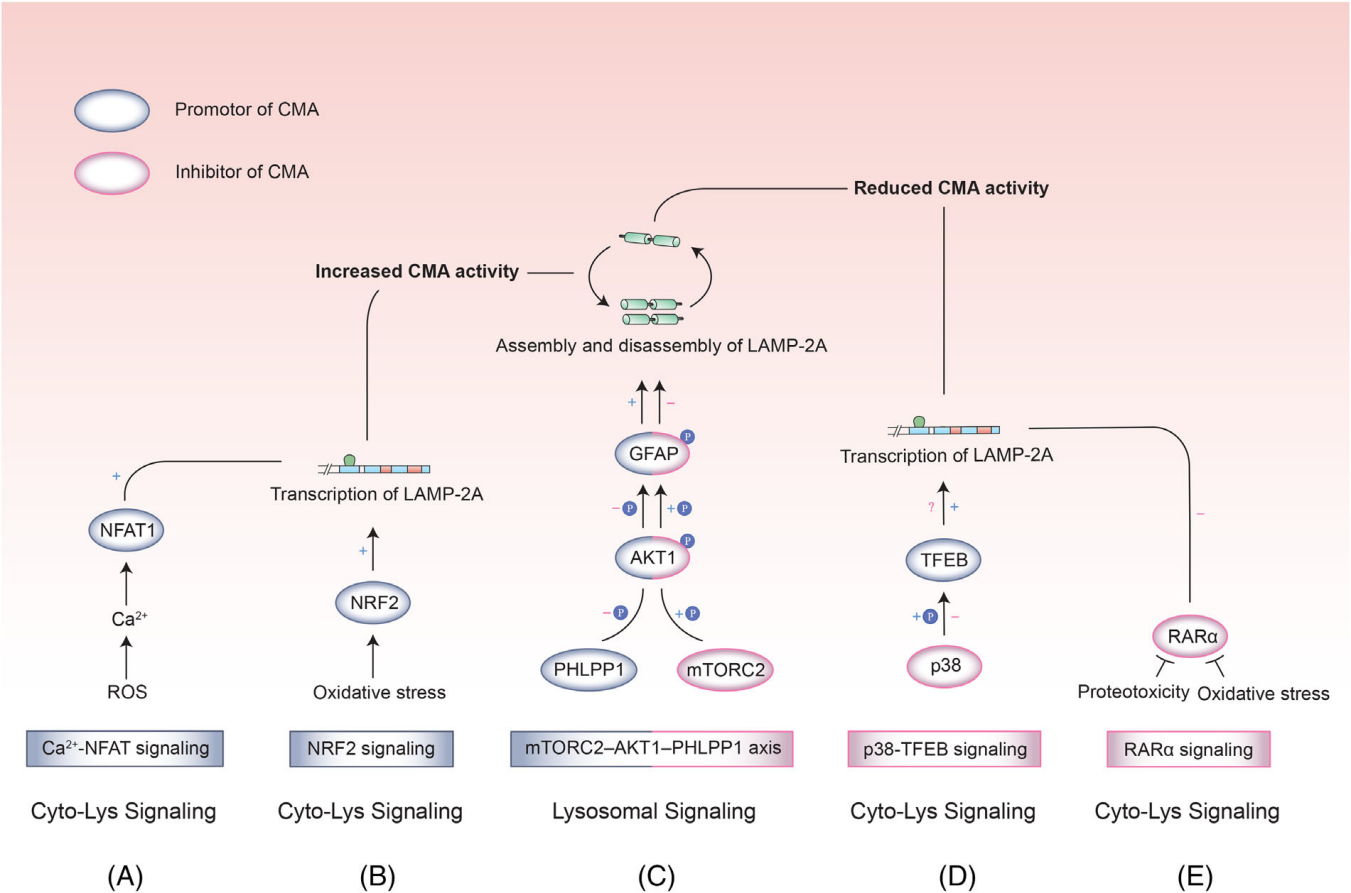
Signaling pathways regulating CMA. (A) Ca^2+^–NFAT signaling: This is the first signaling pathway that was found to modulate LAMP‐2A transcription, which regulates CMA activity in T cell activation and proliferation. ROS stimulates the influx of Ca^2+^ into the cell. Subsequently, NFAT1 directly binds to the promoter of the *LAMP‐2A* gene, leading to an increased transcription of *LAMP‐2A* and CMA activity. (B) NRF2 signaling: Under oxidative stress, NEF2 binds to the ARE site in LAMP‐2A to promote transcription and expression of LAMP‐2A. This promotion can be achieved with pharmacological activation of NRF2 and is destroyed when NRF2 knockdown is performed in the liver. (C) TORC2–AKT1–PHLPP1 axis: This axis plays a dual role in the regulation of CMA. PHLPP1 dephosphorylates AKT1, and dephospho‐AKT1 promotes GFAT dephosphorylation. Dephospho‐GFAT promotes the assembly and disassembly of LAMP‐2A, thus boosting CMA activity. In contrast, TORC2 phosphorylates AKT1, therefore promoting phosphorylation of GFAP, and increasing CMA activity. (D) p38–TFEB signaling: The MAPKs family of serine/threonine kinases, p38, can phosphorylate TFEB to inhibit its activity, however, there are conflicting reports on whether TFEB regulates CMA by promoting LAMP‐2A transcription. (E) RARα signaling: This signaling inhibits CMA activity by decreasing the expression of LAMP‐2A, RAB11, and RILP. LAMP‐2A limits the rate of CMA, and Rab11 protein and RILP facilitate LAMP2A trafficking. RARα inhibitor QX77 was found to release the inhibition of CMA activity, therefore protecting cells against proteotoxicity.

So far, five signaling pathways were introduced to involve in the regulation of CMA (Figure 6). Three of these pathways were revealed earlier and have been widely accepted, while NRF2 and p38–TFEB are the most recently uncovered CMA regulatory pathways. It is much more difficult to study the regulatory pathways compared to finding new autophagic substrates. Since the signaling molecules may act in multiple pathways rather than specifically targeting autophagy. To make matters worse, there are very few studies on CMA compared with the heavily studied regulatory pathways of macroautophagy. As mentioned above, CMA has only been found in animals such as mammals and birds and is not observed in simpler organisms such as yeast and *Drosophila*, posting an obstacle to studying protein interactions using in vitro models.^26^

### 5.1 Ca^2+^–NFAT signaling

This is the first signaling pathway that was found to modulate LAMP‐2A transcription, which regulates CMA activity in T cell activation and proliferation. After T‐cell activation, TCR‐induced ROS production stimulates the influx of Ca^2+^ into the cell. Subsequently, NFAT1 directly binds to the promoter of the *LAMP‐2A* gene, leading to an increased transcription of *LAMP‐2A* and CMA activity.^35^ Enhanced CMA activation reduces two inhibitors of T cell activation, Rcan‐1, and Itch, thus ensuring the functional integrity of T cell activation, proliferation, and differentiation.^85^, ^87^, ^117^ During T‐cell activation, Ca^2+^–NFAT signaling functions as “positive feedback,” in which activated T cells upregulate CMA and then CMA degrades negative regulatory molecules to maintain T‐cell activation.

### 5.2 mTORC2–AKT1–PHLPP1 axis

This axis exerts a dual role in the regulation of CMA via acting on AKT1, the shared downstream molecules of mTORC2 (target of rapamycin complex 2) and PHLPP1 (pleckstrin homology domain leucine‐rich repeat protein phosphatase 1).^118^ Different from the other four pathways, the mTORC2–AKT1–PHLPP1 axis is a solely lysosome‐dependent signal without the involvement of cytoplasmic signals. The intact signaling pathway can be identified from lysosomes isolated from cells, which demonstrates the independence of lysosomal signaling.^118^ Among them, mTOR constitutes two protein complexes, mTORC1, and mTORC2, involved in the regulation of cell growth, metabolism, survival, and proliferation.^119^, ^120^ Under nutrient sufficiency, mTORC1 negatively regulates macroautophagy via the inhibition of two macroautophagy complexes (unc‐51 like autophagy activating kinase [ULK] and vacuolar protein sorting [VPS]34).^121^ While in CMA, the mTORC in charge of regulation is mTORC2, which localizes to the lysosomal membrane and phosphorylates downstream AKT1, thus promoting the glial fibrillary acidic protein (GFAP) phosphorylation.^118^, ^122^, ^123^ This process stabilizes GFAP to enable its separate existence, prevents GFAP from binding to the LAMP‐2A–HSC70 complex, promotes dissociation of LAMP‐2A, and therefore exerts an inhibitory effect on CMA.^124^ In contrast, PHLPP1 dephosphorylates AKT1, and dephospho‐AKT1 promotes GFAT dephosphorylation. Dephospho‐GFAT promotes the assembly and disassembly of LAMP‐2A, thus boosting CMA activity.^118^ Incidentally, in macroautophagy, AKT phosphorylates downstream effectors Beclin‐1 and FOXO3, which inhibit macroautophagy by suppressing autophagosome formation. Therefore, AKT might emerge as one of the cosignaling molecules for the interplay between macroautophagy and CMA, although this has not been validated in any study to date.^125^

### 5.3 Retinoic acid receptor alpha signaling

In macroautophagy, retinoic acid exerts positive modulation by promoting autophagosome maturation, upregulating Beclin‐1, and downregulating mTOR.^126^, ^127^, ^128^ While in CMA, retinoic acid receptor alpha (RARα) signaling is a negatively regulated pathway. RARα knockdown resulted in increased protein degradation in fibroblasts which was inhibited by CMA inhibitors but not by macroautophagy inhibitors, thus confirming that CMA is a lysosomal degradation pathway that is regulated by RARα signaling. In contrast, the CMA degradation pathway was significantly damaged in RARα (+) wild‐type cells or cells with the addition of ATRA (receptor agonist of RARα).^129^ This signaling inhibits CMA activity by decreasing the expression of LAMP‐2A, Rab11, and RILP. LAMP‐2A limits the rate of CMA, and Rab11 protein and Rab‐interacting lysosomal protein (RILP) facilitate LAMP2A trafficking. RARα inhibitor QX77 was found to release the inhibition of CMA activity, therefore protecting cells against proteotoxicity and oxidative stress. Furthermore, the promotion of CMA by inhibiting the RARα receptor will be accompanied by a downregulation of macroautophagy activity, which may reflect the interaction between the two lysosomal degradation pathways.^129^

### 5.4 NRF2 signaling

As it was mentioned above, degradation of Keap1 by CMA can maintain the stability of NRF2 under oxidative stress, and subsequently, NRF2 binds ARE of target proteins (including phase II metabolic enzymes, antioxidant proteins, etc.) to promote the expression of antioxidant molecules.^57^, ^58^ It was found that NRF2 itself not only receives indirect regulation by CMA through Keap1 but also directly modulates CMA levels. At least two ARE‐binding sites were identified in *LAMP‐2A* that could combine with NRF2 to boost *LAMP‐2A* transcription. Pharmacological activation of NRF2 promoted LAMP‐2A protein expression and elevated CMA activity. In the NRF2 knockout hepatocyte model, oxidative stress failed to increase LAMP‐2A levels.^80^ These findings enrich the understanding of NRF2 substrates and introduce a new family member to the regulation of the NRF2–Keap1–ARE signaling.

### 5.5 P38–TFEB signaling

P38–TFEB signaling was discovered in the investigation of NLRP3 signaling in Parkinson's disease (PD). In the mouse model of PD, α‐synuclein accumulation was accompanied by activation of NLRP3 inflammasome to involve in the pathophysiology of PD.^130^ In microglia, TFEB enhances the degradation of NLRP3 by increasing the protein level of LAMP‐2A, whose activity is regulated by p38.^79^ P38 is a serine/threonine kinase of the mitogen‐activated protein kinases (MAPKs) family and phosphorylates TFEB to inhibit its activity.^131^ P38 inhibitors could increase the translocation of TFEB from the cytoplasm to the nucleus, and ultimately facilitates NLRP3 degradation via the lysosomal pathway, reducing α‐synuclein levels in PD mice and attenuating neuroinflammation caused by abnormal protein accumulation.^79^ In ovarian cancer cells, TFEB was also revealed to augment mRNA and protein levels of LAMP‐2A.^81^ However, the mechanism by which TFEB promotes LAMP‐2A transcription and expression is not yet clear, and previous studies have argued that LAMP‐2A is not subject to transcriptional regulation by TFEB.^129^, ^132^

## 6 CMA AND HUMAN DISEASES

Since CMA is involved in the maintenance of normal physiological functions, CMA deficiency may lead to diseases. Actually, CMA activation is engaged in the pathological processes or protective mechanisms of some diseases (Table 1). Here, we have included two of the more widely studied disorders (NDs and cancer), as well as cardiovascular disease and intestinal disorders that have not been well described in other reviews, while in which an increasing number of investigations have revealed the involvement of CMA.

**TABLE 1:** CMA in human diseases.

Disease	CMA activity	Regulation mechanism	CMA target	CMA effects	Observations	References
Acute liver failure	Increased	PI3K/AKt/mTOR, negative	Unknown	Protective	Inhibition of PI3K/AKt/mTOR protects against acute liver failure via CMA	^133^
Acute kidney injury	Increased	Asparaginyl endopeptidase legumain, positive	GPX4	Pathogenic	CMA of GPX4 facilitates tubular ferroptosis in AKI	^134^
Alcoholic liver steatosis	Reduced	SNX10, positive	Unknown	Pathogenic	SNX10 mediates alcoholic liver steatosis via activation of CMA	^135^
Alzheimer's disease	Reduced	Unknown	α‐Syn, tau	Protective	CMA prevents the aggregation of α‐syn and tau in AD	^42^
Atherosclerosis	Reduced	Aging or excessive lipid feeding, negative	NLRP3	Protective	Deficient CMA inhibited NLRP3 inflammasome degradation to promote atherosclerosis progression	^136^
Cancer	Increased	Unknown	TCTP (acetylated), CIP2A, MDM2, P65	Protective^a^	CMA reduces levels of prooncogenic proteins	^137^
	Increased	Tumor microenvironment (partly regulates), positive	Antiproliferative protein RND3, tumor suppressors such as MST1, PED and mutant p53, proapoptotic protein BBC3/PUMA	Pathogenic^a^	Upregulation of CMA eliminates antitumor proteins	^137^
	Reduced	Unknown, negative	AF1Q	Pathogenic^a^	Downregulation of CMA leads to high expression of AF1Q, which proposes a proto‐oncogenic role and participates in the regulation of apoptosis in pediatric AMI	^137^, ^138^
Cystinosis	Reduced	CTNS, positive	Unknown	Protective	CTNS deficiency leads to CMA impairment in cystinosis, which features cystine accumulation	^139^
Diabetes	Reduced	Growth factors including EGF, negative	Pax2	Protective	Reduced pax2 degradation via CMA leads to diabetic‐induced renal hypertrophy	^140^
Diabetic retinopathy	Reduced	GMFB, negative	ACSL4	Protective	Accumulation of ACSL4 induces ferroptosis in RPE cells	^141^
Heart failure	Reduced	Presenilin‐2 (an intramembrane protease), positive	RyR2 (after oxidative damage)	Pathogenic	CMA may target oxidative RyR2 to promote the survival of cardiomyocytes	^16^
Hepatitis C	Increased	HCV NS5A protein, positive	HNF‐1α	Pathogenic	CMA involves in the degradation of HNF‐1α in HCV‐infected cells, which may promote HCV replication	^142^, ^143^
Huntington's disease	Reduced	Unknown	Mutant N‐terminal huntingtin protein	Protective	Huntington's disease features the accumulation and aggregation of mutant Huntington's protein, which is a substrate for CMA	^144^
Ischemic heart disease	Increased	Hypoxia, positive	Unknown	Protective	LAMP‐2A overexpression reduced hypoxia‐induced apoptosis by 50%	^145^
Lupus	Increased	HSPA8/HSP90AA1, positive	Unknown	Pathogenic	Inhibition of CMA alleviates autoimmunity in lupus	^146^
Mucolipidosis type IV	Reduced	TRPML1, positive	Oxidized proteins (hypothesis)	Pathogenic	Impairment of CMA results from reduced expression of LAMP‐2A was found in mucolipidosis type IV fibroblasts	^147^
Nonalcoholic steatohepatitis	Reduced	Unknown	Unknown	Pathogenic	CMA may involve in lipid accumulation and hepatocellular injury via regulation of lipid metabolism and immune responsiveness (hypothesis)	^148^
Parkinson disease	Reduced	Unknown	Aberrant α‐synuclein	Protective	α‐Synuclein mutant block its degradation via CMA, leading to α‐synuclein accumulation in the Lewy inclusion bodies	^108^
Spinal cord injury	Increased	HDAC6, positive	ROS damage proteins	Protective	CMA prevents oxidative damage in injured neurons after spinal cord injury	^60^, ^149^
Traumatic brain injury	Increased	Unknown	Cellular aberrant proteins	Protective	Microglia proliferation in injured area is observed after upregulation of CMA activity	^150^, ^151^
Closed head injury	Increased	Sirt1, positive	Damage proteins in astrocytes	Protective	Increased CMA ameliorated neurological deficits, reduced tissue loss, and attenuated astrocyte activation after CHI	^151^
Ulcerative Colitis	Increased	Unknown	Unknown	Pathogenic	Inhibition of CMA leads to an ameliorated disease progression of ulcerative colitis	^152^

ACSL4, acyl‐CoA synthetase long‐chain family member 4; AD, Alzheimer's disease; AF1Q, ALL1 fused gene from chromosome 1q; AKI, acute kidney injury; AML, acute myeloid leukemia; α‐syn, α‐synuclein; CIP2A, cancerous inhibitor of protein phosphatase 2A; CTNS, cystine transporter cystinosin; BBC3/PUMA, BCL2 binding component 3; HSP, heat shock protein; GMFB, glia maturation factor‐β; HCV, hepatitis C virus; HNF‐1α, hepatocyte‐nuclear factor 1α; MDM2, mouse double minute 2; MST1, mammalian STE20‐like kinase‐1; NLRP3, NLR (NOD‐like receptor) family, pyrin domain containing 3; PED, phosphoprotein enriched in diabetes; RND3, Rho family GTPase 3; ROS, reactive oxygen species; RPE cells, retinal pigment epithelial cells; RyR2, ryanodine receptor type 2; SNX‐10, sorting nexin −10; TCTP, translationally controlled tumor protein; TRPML1, transient receptor potential mucolipin‐1.

^a^CMA has dual functions in cancer. Physiologically, CMA protects normal cells against malignant transformation and tumorigenesis. Loss of CMA contributes to steatosis, genomic instability and decreased prooncogenic protein quality control, which favor malignant transformation and tumorigenesis in liver. In cancer cells, CMA protects them from ER stress and hypoxia, sustains energy metabolism, and contributes to proliferation, invasion and migration.^137^

### 6.1 Neurodegenerative disorders

NDs are a class of age‐related, degenerative diseases with specific neuronal damage.^153^ Among them, Parkinson's disease, AD, and HD are three typical NDs with the common feature of abnormal protein aggregation, by which CMA involves in disease protection or pathogenesis.

#### 6.1.1 Parkinson's disease

Characterized by resting tremor and bradykinesia, PD is a common middle‐aged ND.^154^ Loss of dopaminergic neurons and the formation of eosinophilic inclusion bodies (i.e., Lewy bodies) with α‐synuclein as the main component constitute the major pathological features of PD.^155^ The pathogenesis of PD is still unclear and may be associated with genes, environment, and epigenetics.^156^ Among them, α‐synuclein regulated by more than 20 genes, represented by *PARK* family genes, has always been an interesting protein target. It is well established that multiple genetic mutations lead to the accumulation of α‐synuclein, which is involved in the pathogenesis of PD.^157^, ^158^

PD is the first human disease identified to be associated with CMA dysfunction.^108^ CMA impairment was confirmed in brain tissue specimens and body fluids of patients with PD. LAMP‐2A and HSC70 levels are lower in the brain substantia nigra and amygdala of PD patients than in their non‐PD counterparts.^159^ Although LAMP‐2A levels did not differ between PD and healthy individuals in peripheral blood mononuclear cells, reduced levels of HSC70 were detected in PD patients, which may indicate that decreased levels of key regulatory proteins of CMA are presented not only in brain tissue but also in body fluids.^160^

CMA defects are mediated by mutant variants and are involved in PD pathogenesis by reducing the degradation of the deleterious protein α‐synuclein. To date, five of the PD‐associated pathogenic protein variants have been found to potentially interfere with CMA function, some of which serve as CMA substrates while others do not.

α‐Synuclein is encoded by *SNCA* (α‐synuclein) (also known as *PARK1*), of which wild type exists in an oligomeric form and is degraded by mitochondrial autophagy and CMA. Mutations or posttranslational modification errors of *SNCA* can mediate the formation of α‐synuclein aggregates.^161^ α‐synuclein accumulation acts as both the cause and consequence of CMA dysfunction. In vitro, two mutants of α‐synuclein (A53T and A30P) bind tightly to LAMP‐2A but are incapable of internalization, and the consequent CMA deficiency also blocks the lysosomal pathway degradation of other molecules including GAPDH.^108^ Similarly, phosphorylation and dopamine‐modified α‐synuclein also block the internalization of substrates and CMA degradation of other protein substrates.^162^ In the microglia of the PD model, the α‐synuclein A53T mutant was found to activate the p38 TFEB pathway and inhibit CMA degradation of NLRP3 inflammasomes.^79^

The ubiquitin C‐terminal hydrolase L1 (UCH‐L1) mutant itself is primarily subject to degradation by macroautophagy and is not a substrate for CMA. However, UCH‐L1 can directly interact with LAMP‐2A, Hsc70, and Hsp90 to inhibit CMA activity and ultimately mediate α‐synuclein accumulation. However, the detailed mode of interaction of these proteins has not been revealed.^163^ Vacuolar protein sorting‐35 (VPS35) is also a non‐CMA substrate, of which mutation or deficiency can prevent Golgi recycling of LAMP‐2A and promote LAMP‐2A degradation.^164^

Abnormal LAMP‐2A‐stained lysosomal nuclear aggregation was observed in the striatum of mice with mutations in *LRRK2* (leucine‐rich repeat kinase‐2) (also known as *PARK8*), the pathogenic gene of PD, suggesting impaired degradation of abnormal proteins by the lysosomal pathway.^165^, ^166^ Despite increased levels of LAMP‐2A and HSC70 in the striatal lysosomal membranes of aged mice, which may reflect upregulated CMA activity, the levels of the CMA substrate GAPDH were increased in neurons in this region. In *LRRK2* mutant mouse fibroblasts, reduced clearance of KFERQ motif‐containing proteins was also observed, and the application of RARα antagonists activated impaired CMA under this condition, increasing lysosomal activity.^165^ Surprisingly, protein levels of LAMP‐2A and HSC70 were not significantly raised after activation of CMA despite elevated LAMP‐2A mRNA levels, suggesting that LAMP‐2A levels in the presence of gene mutations may not accurately respond to CMA activity or that *LRRK2* mutations may mediate CMA defects in a manner other than LAMP‐2A and HSC70.^165^, ^167^

CMA function is not only disrupted by these cytoplasmic proteins mentioned above but may also be blocked by noncytoplasmic proteins. Misfolded β‐glucocerebrosidase, encoded by mutant alleles of *GBA1*, fails to be translocated from the endoplasmic reticulum into the lysosome, where it is recognized by HSC70 and bound to LAMP‐2A, preventing CMA from degrading α‐synuclein and Tau. These changes lead to abnormal protein aggregation in mouse nigrostriatal neurons and thus mediate neurotoxicity.^168^

The abnormal protein encoded by the mutant gene occupies the LAMP‐2A site in the lysosomal membrane or restricts the CMA process, thereby preventing CMA from degrading various protein substrates, including α‐synuclein, an important contributor to PD pathogenesis.^108^ The accumulation of wild‐type α‐synuclein has been reported to be sufficient to cause human PD, and the upregulation of macroautophagy following impairment of the CMA pathway may represent a form of compensation, albeit a very limited one.^169^ It is necessary to note that in the pathophysiology of PD, complex factors involving abnormal protein aggregation, oxidative stress damage, inflammatory cell activation, and mitochondrial dysfunction are intertwined, and CMA is coregulated with the ubiquitination‐proteasome pathway, a complex process that requires further study and clarification.^156^ Therefore, this intricate process warrants further clarification. For instance, CMA may also engage in PD pathogenesis by mediating mitochondrial damage. Oxidative damage in PD decreases CMA activity, mediating the accumulation of the E3 ubiquitin ligase MARCHF5, which in turn promotes DNM1L translocation and ultimately leads to the hyper division of mitochondria.^170^

Based on the mechanism of CMA involvement in PD pathogenesis, CMA may serve as a therapeutic target for PD. Restoration of CMA function has been reported to attenuate the neurotoxicity associated with wild‐type and variant α‐synuclein.^171^ LAMP‐2A is the most frequently considered target for CMA intervention. The half‐life of α‐synuclein was reduced in cell lines with high expression of LAMP‐2A. Elevated CMA activity exerts a neuroprotective effect regardless of whether the actual level of α‐synuclein is altered.^172^ The introduction of LAMP‐2A into *Drosophila*, where CMA activity was not otherwise present, was found to confer stress resistance and delay aging.^173^ In addition, the transcriptional regulator DJ‐1 (encoded by *PARK7*) resists α‐synuclein accumulation in the early stage of aggregation also attributes to an upregulation of LAMP‐2A levels.^174^, ^175^ Another target of intervention is HSC70, responsible for recognizing substrates and transporting them to the lysosome. Mesencephalic astrocyte‐derived neurotrophic factor (MANF) increases the binding of HSC70 to wild‐type α‐synuclein, thereby promoting its CMA degradation and rescuing dopaminergic neurons. However, the degradation of α‐synuclein (A53T) under this stimulation condition is mediated by macroautophagy rather than CMA, which is also consistent with the poor degradation of mutant α‐synuclein by CMA in other reports.^176^ Beyond this, stimulation of CMA through modulation of signaling pathways is also feasible. A typical case is the addition of RARα antagonist, a CMA activator, to neuronal cell culture medium, which was found to reduce α‐synuclein aggregation in *LRRK2* mutation scenarios, whereas the mechanism of CMA upregulation is not yet defined.^165^

#### 6.1.2 Alzheimer's disease

AD is the most common form of dementia worldwide and is characterized by extracellular Aβ deposition and intracellular tau accumulation. Protein aggregation forms tangles and plaques, ultimately leading to brain cell death and brain atrophy.^177^

CMA deficiency may be involved in the pathogenesis of AD. Among the three autophagic modalities, CMA is the major degradation pathway for wild‐type tau, and inhibition of CMA almost completely eliminates lysosomal degradation of tau.^178^, ^179^ However, although APP also contains the KFERQ motif, neither APP nor Aβ is a substrate for CMA. In hepatocytes and immortalized neuronal cells, the greatest risk factor for AD, apolipoprotein E4 isoform (ApoE4), is degraded by macroautophagy and CMA, with CMA as the more dominant pathway.^180^ In addition, CMA is involved in the degradation of RCAN1, assisting in limiting the activity of calcineurin to prevent excessive tau phosphorylation.^117^, ^181^

AD also interferes with the integrity of the CMA function. Compared to PD, the inhibition of CMA by tau is more complex and varies among variants.^178^ Acetylated tau may inhibit CMA and reduce tau degradation by decreasing its pH sensitivity of binding to HSC70.^179^, ^182^ Tau (FTDP‐17) contains two KEFRQ motifs, cleaved by the proteasome into an F1 fragment that binds to LAMP‐2A of the lysosomal membrane, enters the lysosome with its C‐terminal translocation, and the inserted part is then degraded by lysosomal enzymes into two fragments, F2 and F3. The formation of these fragments prevents the subsequent steps of CMA, limiting tau degradation and the elimination of other substrates through CMA.^183^

Therefore, treatment targeting AD is generally based on the activation of CMA. Currently, drugs including metformin and lactulose have been found to activate CMA and improve AD in mice.^184^, ^185^, ^186^ Another pathway is to modify the substrate to make it more susceptible to degradation by CMA. Juan Dou et al. designed a segment of targeting peptides containing the KFERQ motif and oxidizable amino acids to take advantage of the tendency of CMA to degrade oxides. The targeting peptide binds to Aβ and directs it to the lysosome, where it is degraded by either the CMA pathway or the microautophagy pathway.^187^

However, the future of drug development for AD is uncertain, and a study last year pointed out that neuronal cell death precedes Aβ deposition, which may partly explain the fact that no drugs targeting Aβ plaques have so far been able to exert the desired clinical effect.^188^ Given that the pathogenic effects of Aβ and tau on AD are still controversial and enigmatic, there is still a long way from basic to the clinical application regarding targeted CMA for AD.

#### 6.1.3 Huntington's disease

Compared to PD and AD, the two most common forms of NDs, HD is rare, with an estimated global prevalence of approximately 2.7/100,000 (the year 2012).^177^, ^189^ HD is characterized by the presence of polyQ repeat sequences in Htt mediating protein aggregation and neurotoxicity.^190^

CMA was found to clear mutant Htt such as Htt‐552, with stronger clearance than for wild‐type. This clearance was significantly diminished after inhibition of HSC70 or LAMP‐2A.^191^, ^192^ The elimination of Htt by CMA appears to be less than that of macroautophagy and is age‐dependent. In the early stages of HD, an abnormally prolonged polyQ sequence in mHtt upregulates LAMP‐2A levels and CMA activity increases to clear Htt.^111^ However, as HD progresses, the compensatory effect of CMA on macroautophagy falls.^193^

Similar to PD and AD, the key to CMA‐based alleviation of HD is to improve the degradation of denatured proteins.^194^ Alginate induces the expression of cochaperone HSPB8 (i.e., HSP22) and BAG3 through the TFEB pathway and thus upregulates CMA to treat a variety of NDs including HD.^195^ In addition, analogous to the synthetic peptide for the treatment of AD, the linker that targeted polyQ and two HSC70 binding motifs were combined into a peptide that could be integrated into the repetitive sequence of Htt and guided through CMA degradation, reducing Htt aggregation in the mouse striatum.^196^

### 6.2 Cancer

CMA plays a dual regulatory role in cancer. CMA viability drops with age, which is consistent with the increased incidence of various tumors with age. However, the function of CMA can also be utilized by tumors, thus promoting tumor progression.^137^

CMA regulates the levels of proto‐oncogenes and oncoproteins to exert its oncogenic effects. In the early stage of tumors, CMA can resist malignant transformation caused by proto‐oncogene MYC in normal cells. The cancerous inhibitor of protein phosphatase 2A (CIP2A) is an oncoprotein that inhibits the degradation of MYC and remains its stabilization by inhibiting Ser62 phosphatase in the degradation process. CMA degrades CIP2 which is responsible for the stabilization of MYC, therefore plays a tumor‐suppressive role in normal cells.^197^, ^198^ In CMA‐deficient fibroblasts, it was found that MYC protein accumulates and MYC‐driven malignant transformation was facilitated.^197^ Similarly, translationally controlled tumor protein (TCTP) and murine double minute (MDM2) are also both CMA substrates, of which levels are tightly regulated to maintain proteostasis under normal conditions.^199^, ^200^

CMA also limits lesions with the potential for malignant progression. In the liver, increased LAMP‐2A stability attenuates cirrhosis and liver injury in alcoholic liver disease.^135^ LAMP‐2A knockout mice have a stronger propensity for liver fibrosis confronting oxidative stress, a major pathological process in nonalcoholic liver disease.^201^ These pathological changes point to hepatocellular carcinoma together.^137^ CMA deficiency also reduces the degradation of NF‐κB p65, leading to increased levels of Twist and Snail and the reduced level of E‐cadherin, thus promoting the EMT process, a process critical for tumorigenesis and metastasis.^75^, ^202^, ^203^, ^204^

CMA can be utilized by tumor cells as a contributor to their growth, proliferation, and metastasis. An increasing number of CMA substrates have been identified to be involved in various periods of tumors, and CMA activity is highly expressed in a variety of tumors including prostate cancer, glioblastoma, and ovarian cancer.^205^, ^206^, ^207^ The mechanisms by which CMA promotes tumors are not fully understood, but at least tumors can exploit CMA to regulate metabolism and cell cycle, adapt to stressful environments, and resist apoptosis.^26^, ^208^ During tumor cell growth, CMA degrades PKM2 to accumulate the intermediate products of the glycolytic pathway for cellular anabolism and promoting the growth of tumor cells.^209^, ^210^ The proapoptotic protein BBC3/PUMA is also eliminated via CMA, which resist the apoptosis of tumor cell.^211^ In conclusion, tumors will leverage the ability of CMA to adapt to stress to establish a microenvironment more conducive to their survival.^26^

Besides, CMA is also associated with tumor drug resistance.^212^ In colorectal cancer, five‐fluorouracil (5‐FU) was found to reduce the binding ability of histone acetyltransferase E1A‐binding protein and CREB‐binding protein (p300/CBP) to chromatin and induce its degradation through CMA, thereby inducing histone deacetylation in colorectal cancer cells.^213^ Deacetylation prevents the access of DNA to replication factors, thereby reducing the access of 5‐FU to DNA and limiting the efficacy of 5‐FU.^214^ Based on this, limiting CMA activity may increase the efficacy of antineoplastic drugs. Downregulation of CMA activity has been found to increase the sensitivity of esophageal cancer to cisplatin and non‐small cell lung cancer to cisplatin.^215^, ^216^

Given the role of CMA in tumor prevention and pathogenesis, a CMA‐based therapeutic strategy should be to maintain it at physiological levels. However, although the inhibition of CMA in the tumor‐bearing mice model showed initial benefits, this therapeutic effect needs to be considered in a more complex tumor setting, and more investigations are needed to explain the involvement of CMA in tumor progression.^216^

### 6.3 Intestinal disorders

Emerging studies have revealed associations between CMA and intestinal mucosal immunity and the intestinal barrier leading to diseases. Here, we list the diseases associated with CMA and discuss their pathogenesis and potential targets.

#### 6.3.1 Inflammatory bowel disease

Inflammatory bowel disease (IBD) is a chronic immune‐mediated disease with an increasing incidence that imposes a socioeconomic burden.^217^ The pathogenesis of IBD is closely related to damage to the intestinal mucosal barrier. The mechanisms of IBD include epithelial cell tight junction damage, excessive T cell activation, oxidative stress, and dysbacteriosis. EMT is also involved in intestinal fibrosis in IBD patients. In Crohn's disease, T cells are overactivated and Th1 cells produce excess IL‐12, IL‐17, and IL‐23.^218^ Inhibition of CMA can enhance the inhibitory effect of MSCs on T cells, thereby reducing cytokine damage to the intestinal mucosal barrier. CMA degrades oxidative products under oxidative stress, thereby exerting a protective effect on the intestinal barrier. Moreover, CMA can also inhibit EMT progression by degrading p65 in the NF‐κB signaling pathway. Therefore, CMA serves as both a pathogenic and protective mechanism to IBD.

Recently, CMA was shown to be a potential therapeutic target for ulcerative colitis. In the dextran sodium sulfate (DSS)‐induced colitis model, no obvious changes were detected in LAMP‐2A at the gene level, whereas protein expression of LAMP‐2A was increased. After treatment with the CMA inhibitor P140, the altered expression of LAMP‐2A was attenuated, leading to the amelioration of disease progression.^219^ Although P140 was shown to regulate markers of CMA and ameliorate the symptoms of ulcerative colitis in the DSS model, the mechanism of increased LAMP‐2A, the pattern of CMA involvement in the pathogenesis of ulcerative colitis, and the efficacy of P140 in patients with ulcerative colitis remains to be determined.

However, the roles and treatment of IBD remain to be fully elucidated. CMA might maintain the activation of CD4^+^ T cells to regulate intestinal immunity and protect the intestinal barrier by producing multiple cytokines. Two cytokines, IL‐17 and IL‐22, play complex roles in the pathogenesis of IBD. As upstream mediators of proinflammatory factors IL‐1 and TNF, IL‐17 might induce an inflammatory cascade that aggravates inflammation.^220^ Increased levels of IL‐17 have been found in the mucosa of patients with active IBD.^221^ However, IL‐17 may also play a protective role in the maintenance of the intestinal barrier, as it promotes the production of granulocyte‐macrophage colony‐stimulating factor (GM‐CSF), antimicrobial peptides, and IgA.^35^, ^222^ Inhibition of IL‐17 weakens the epithelial barrier function and exacerbates IBD.^223^, ^224^ Elevated IL‐22 levels have also been detected in IBD patients.^225^ In active Crohn's disease, IL‐22 promotes intestinal epithelial cell migration and mucosal healing via phosphatidylinositol 3‐kinase‐dependent mechanisms and the production of β‐defensin‐2.^226^ In models induced by dinitrobenzene sulfonic acid and anti‐CD40, IL‐22 plays a disease‐promoting role.^227^, ^228^ The complex cytokine network remains a challenge that requires further exploration.

#### 6.3.2 Intestinal fibrosis

Intestinal fibrosis occurs in various intestinal diseases, including IBD, carcinogenesis, invasion of colorectal cancer, and radiotherapy.^229^, ^230^, ^231^ As a stage of chronic inflammation, intestinal fibrosis is a frequent complication of IBD, causing intestinal strictures and bowel obstruction, which leads to an increased surgical rate and deteriorating prognosis.^232^ In contrast to its dual effects on pathogenesis and protection of intestinal inflammation, CMA inhibits EMT progression against intestinal fibrosis by downregulating NF‐κB (p65/RelA) signaling.^75^ Rigosertib, an inhibitor of NF‐kB (p65/RelA), was found to ameliorate intestinal fibrosis in DSS‐induced colitis mice.^233^ As emerging therapies have been developed to treat intestinal fibrosis, and the efficacy of existing medications requires further investigation, CMA might serve as a potential target for the inhibition of EMT progression.

Nevertheless, compared with macroautophagy, which has been proven to be associated with intestinal mucosal immunity and the intestinal barrier, little was found in the direct link between CMA and intestinal homeostasis, and the elaboration of this section on CMA and intestinal disorders require to be confirmed by further studies.

### 6.4 Cardiovascular disease

The protective effect of CMA against atherosclerosis was recently established. VSMCs and macrophages in mice with advanced atherosclerosis displayed significantly lower CMA activity.^15^ In carotid tissue with unstable plaques and tissues with secondary vascular events, LAMP2 levels were lower.^15^, ^234^ CMA protects the organism against atherosclerosis by regulating lipid metabolism, preventing the dedifferentiation of VSMCs, and regulating the proinflammatory phenotype of macrophages.^15^

The protective effect of CMA may also be related to the degradation of NLRP3 inflammasomes. In the LAMP‐2A knockout mouse model, NLRP3 activation was enhanced, and atherosclerosis in the aorta was accelerated. In contrast, the restoration of LAMP‐2A levels was accompanied by a decrease in NLRP3 levels. Thus, in atherosclerosis, impaired CMA may mediate the pathological process of the disease by reducing the degradation of NLRP3 inflammatory vesicles.^136^

Rescue of CMA function may represent one of the targets against atherosclerosis plaque formation. Indeed, CMA‐elevated mice showed better resistance to confronting the atherosclerosis challenge, as manifested by reduced cholesterol, triglycerides, proinflammatory factors, and diminished plaque.^15^

## 7 DISCUSSION

Here, we reviewed the process of CMA and described its physiological role, regulatory signaling, and its relationship to human diseases. In the last three decades since CMA was discovered, studies have revealed its role in physiological regulation and pathology, deepening the understanding of protein quality control and lysosomal function. CMA is a promising potential target, as its selectivity for degradation substrates may aid in the degradation of specific proteins. In addition to its application in therapy, constitutive upregulation or downregulation of CMA may indicate variations in the disease course and thus may serve as a marker for disease progression or improvement. HSP90A, a cochaperone, may respond to CMA activity and is involved in the maintenance of chondrocyte homeostasis. Recently, a prospective cohort study demonstrated that reduced HSP90A in blood and cartilage tissue was associated with a higher risk of developing osteoarthritis, suggesting that reduced CMA levels may predict a higher incidence of osteoarthritis.^235^

The role of CMA has been highlighted in many studies, but it is important to note that protein degradation in many cases is the result of multiple pathways working cooperatively, and the status of the CMA effects in each of these contexts needs to be properly viewed. A typical case is the modification of NRF2 by ubiquitination, macroautophagy, and CMA in oxidative stress, but it is not clear whether these three pathways interact synergistically, complementarily, or competitively.^56^ In addition, CMA is commonly knocked down in specific organs or tissues in order to investigate the outcomes caused by its degradation of specific substrates, yet may neglect the effects of the resulting degradation of other substrates.

The translation of CMA from cellular intervention to clinical application is difficult. In the case of AD, for example, different tau mutants interfere with the three autophagic pathways in different ways, which may result in different intervention strategies.^178^ It becomes even more complicated when these scenarios occur in the human brain. Modulation based on CMA targets is also challenging as chaperones are not exclusive to CMA and it is difficult to specifically modify CMA in a particular physiological process. Currently, for the regulation of CMA, the development of inhibitors is more difficult compared to agonists, and experimental knockdown is the common approach, which confined its applications to cells or animal models. Recently, the first chemical inhibitor targeting CMA, Polyphyllin D, a small molecule inhibitor of LAMP‐2A interaction with HSC70, was identified to inhibit CMA and suppress tumor growth in non‐small cell lung cancer in vitro. Moreover, Polyphyllin D inhibited STX17–SNAP29–VAMP8 signaling and prevented compensatory upregulation of macroautophagy.^236^ In addition to the application of CMA inhibitors or agonists, the modification of targeted substrates is an innovative attempt. Since macroscopic modulation of CMA may affect the degradation of substrates necessary for physiological conditions, target modification of proteins to render them more suitable for CMA could be considered. With the ability to bind with high affinity to the substrate and be degraded by CMA, the targeting peptide can direct the substrate into the lysosome for degradation. Multiple ligands can also be introduced simultaneously to allow the substrate degraded by multiple pathways.^187^, ^196^ This technique is reminiscent of the proteolysis‐targeting chimeras technology that has recently reached clinical trials, where linkers connect E3 ubiquitin ligases and target proteins to degrade oncoproteins through the ubiquitin–proteasome system.^237^, ^238^

In conclusion, the importance of CMA in physiological activities and disease progression is gradually being recognized. Further studies are still needed to reveal the exquisite regulation of this lysosomal pathway in different tissues and pathophysiological situations to lay the foundation for the development of future targeted therapies.

## AUTHOR CONTRIBUTION

Yao Ruchen drafted the article. Shen Jun designed, drafted, and revised it critically for intellectual content. Both the authors have read and approved the final manuscript.

## CONFLICT OF INTEREST STATEMENT

The authors declare that they have no conflict of interest.

## ETHICS STATEMENT

Not applicable.

## Data Availability

Not applicable.
